# Types of trematodes infecting freshwater snails found in irrigation canals in the East Nile locality, Khartoum, Sudan

**DOI:** 10.1186/s40249-016-0108-y

**Published:** 2016-02-25

**Authors:** Nidal A. I. Mohammed, Henry Madsen, Abdel Aziz A. R. M. Ahmed

**Affiliations:** Department of Zoology, Khartoum College of Medical Sciences, Khartoum, Sudan; Parasitology and Aquatic Diseases, Department of Veterinary Disease Biology, Faculty of Health and Medical Sciences, University of Copenhagen, Frederiksberg, Denmark; Education Expert Company, Riyadh, Kingdom of Saudi Arabia

**Keywords:** Freshwater snails, Trematode cercariae, *Biomphalaria*, *Bulinus*, East Nile locality, Khartoum, Sudan

## Abstract

**Background:**

The planorbid freshwater snails of the two genera, *Biomphalaria* and *Bulinus* -have been vigorously studied due to the role they play as intermediate hosts of schistosomiasis. In Sudan specifically, most studies have focused on the chemical and ecological control of the two genera, but few studies have looked at their biological control. This study explored the coexistence of other species of freshwater snails and the two genera along with their trematode infections in relation to a number of environmental factors in the East Nile locality, Khartoum state, Sudan.

**Methods:**

Freshwater snails from irrigation canals (*abueshreens*) were sampled monthly from January 2004 to December 2005. The snails were examined for trematode infections by cercarial emergence immediately after collection and then weekly for an additional four weeks to allow for the maturation of prepatent infections. Vegetation cover in the study sites as well as the physicochemical characteristics of the water, including temperature, were also recorded.

**Results:**

A total of 10,493 snails, representing seven species, were collected. The most abundant species was *Biomphalaria pfeifferi*, representing 48.6 % of the sample*.* Overall, 14.1 % of the snails were found to be shedding some type of cercariae. Five species were found to have infections; among these the *Bulinus truncatus* species was found to be the most heavily infected, with an overall prevalence of 46.2 %. Double infections were recorded in only two *B. truncatus* snails and one *Cleopatra bulimoides* snail. Twenty different morphotypes of cercariae were recorded, seven of which appeared not to conform to previously described cercariae from Africa. Xiphidiocercariae type 1 was the most common type of cercariae recovered, accounting for 44.3 % of all infections. The density of snails tended to be lower during the summer months than the winter months, except for *M. tuberculata* snails, which were not affected by seasonal changes.

**Conclusion:**

The findings of this study indicate that besides schistosomes, other larval trematodes are found, and some use the same intermediate hosts as the schistosomes. Further studies should be conducted to determine whether some of these trematodes could be manipulated for the biological control of schistosomiasis.

**Electronic supplementary material:**

The online version of this article (doi:10.1186/s40249-016-0108-y) contains supplementary material, which is available to authorized users.

## Multilingual abstracts

Please see Additional file 1 for translations of the abstract into the six official working languages of the United Nations.

## Background

Freshwater snails receive considerable attention as they are intermediate hosts of several trematodes that cause diseases in humans and domestic animals. In Sudan, these are most notably schistosomiasis and fascioliasis. Whereas many studies have been conducted on intermediate host snails, most of these have focused on the specific intermediate host species infected with particular trematode. However, relatively few studies have dealt with the total snail and trematode fauna even though it has been suggested that other snail and trematode species may significantly affect transmission patterns of the aforementioned diseases [1, 2]. In Sudan specifically, research has been done on the epidemiology and distribution of *Schistosoma mansoni* [3–9], *S. haematobium* [9–13], *S. bovis* [14, 15] and *Fasciola gigantica* [16, 17]. Likewise, some attention has been given to the control of their snail intermediate hosts [18–23], but no studies have been undertaken on snail abundance and host relationships of other trematode species, which are either not of medical and/or veterinary importance.

Therefore, the present longitudinal study followed seasonal density fluctuations in populations of freshwater snails and determined the prevalence of associated trematode infections in relation to various environmental factors, in the East Nile locality of Khartoum state, Sudan.

## Methods

### Study area

**Fig. 1:**
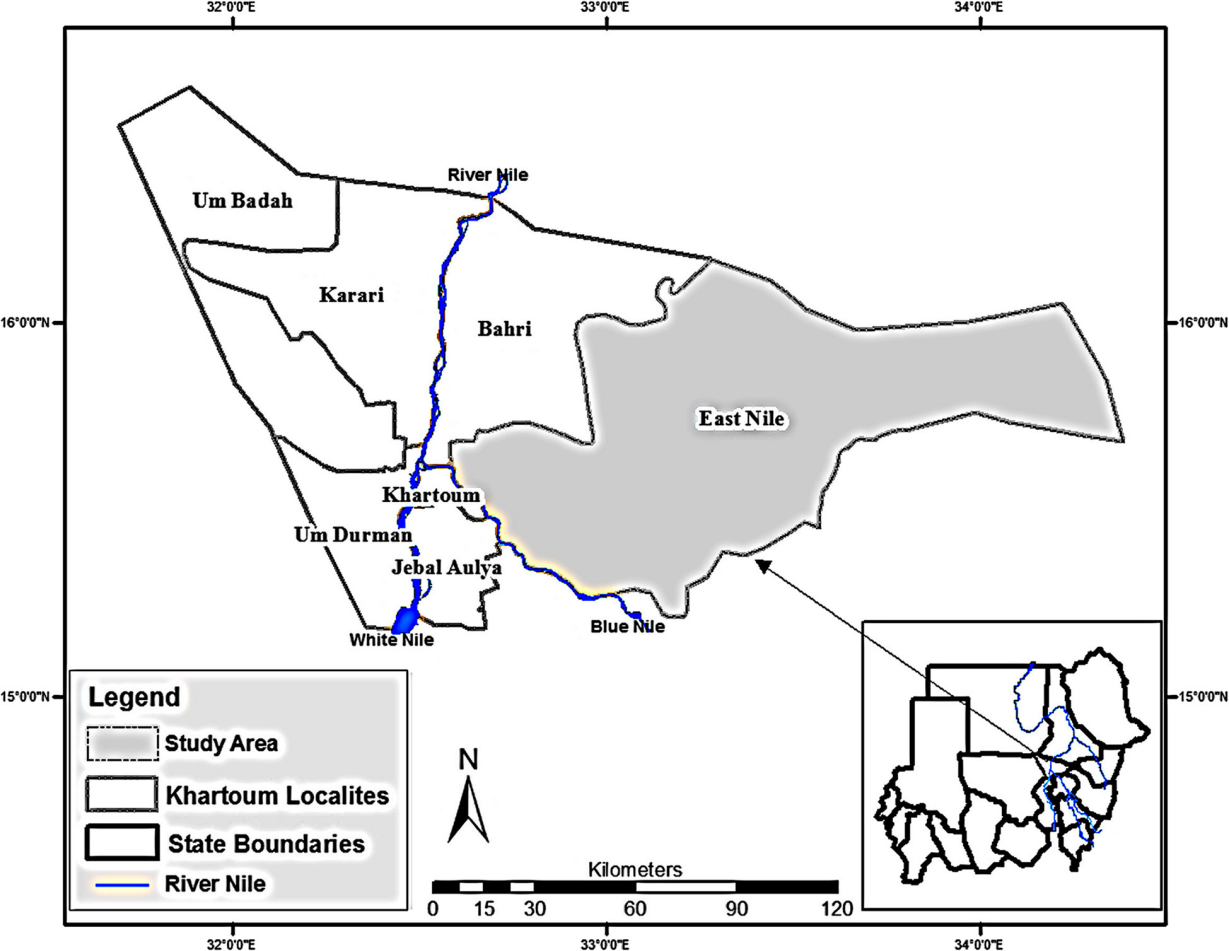
Map showing the study area in the East Nile locality, as well as other localities of Khartoum state, Sudan

The study area chosen is situated in the East Nile locality, an area of about 8000 km^2^ located in the north-eastern part of Khartoum state, Sudan (see Fig. 1). Even though Khartoum is the smallest state in the country by area (22,142 km^2^), it is the most populous (5,274,321 in 2008 census). The state is geographically divided into blocks (or clusters), which are further subdivided into localities. There are a total of three blocks and seven localities. Khartoum has a semi-arid climate, where the rainfall is usually 150–250 mm per year. The mean monthly temperature varies from 25 °C in December to 45 °C in May. The River Nile flood season coincides with the rainy season, peaking in August. Due to low rainfall and the short rainy season, agriculture is mainly of the irrigated type. Agricultural schemes are distributed along the banks of the River Nile and its tributaries; 50 % of the cultivated areas are in the East Nile locality.

There are two seasons in Sudan: summer (May to September) and winter (October to April). Rain falls during the summer season from June to September, which is followed by a long dry season, extending from October to May [24].

### Study design and sampling techniques

Snails and trematodes were collected monthly from January 2004 to December 2005 from canals of an irrigation system of the Blue Nile River, in the East Nile locality, Khartoum. Sampling of the snails was carried out using a scoop made of an iron frame supporting a wire mesh. Environmental and physical factors assumed to affect the presence of snails were recorded i.e. macrophyte cover and algal mass were scored visually and recorded as percentages of the area covered within the sampling sites. Temperature was measured using a mercury thermometer graduated from 0° to 50°. Water depth was measured using a hard wooden ruler. Current velocity was measured by timing a floating object over a two -meter stretch. Shedding of trematode cercariae was conducted by traditional techniques (snails were exposed to artificial light).

### Selection of habitats sampled

The agricultural flat clay land that was included in the study is about 1600 km^2^, with around four major agricultural projects. The study was carried out in irrigation canals (*abueshreens*) of two important agricultural schemes: the southern part of the Al Seilate scheme and the Arabian Company (both irrigate from the Blue Nile). The main cultivated crops are different types of vegetables and fruits, as well as animal fodder. Water-contact activities of farmers and villagers lead to them being exposed to infection with snail-transmitted diseases, especially schistosomiasis, which is considered a major health problem in this area [9, 25, 26].

Three sampling sites, each 200-meter stretches of different irrigation canals (*abueshreens*) characterized by muddy substrata were selected for snail sampling. Site selection was based on: (1) the site’s close proximity to dwellings; (2) preliminary field observations proving the site supports snails, has slow water current, and the presence of aquatic vegetation; and (3) if there is easy access to water for villagers and livestock. Minor canals were not included because they have high water current that do not support snails habitats, and they are not used by villagers because the edges of their banks have dense vegetation making access difficult. Lower order canals such as *abusittas* and *gadwalls* only intermittently contain water for short periods of time.

### Sampling and identification of snails

Monthly collection of snails was conducted from January 2004 to December 2005. Sampling was carried out using a scoop made from a wire mesh measuring 1.5 mm, supported on an iron frame (40 x 30 cm), and mounted on a 1.5 meter long iron handle [23]. One of the authors (NAIM) carried out the sampling of snails to ensure for a standardized sampling effort. Sampling was usually performed between 8:30 am and 10:30 am, for 15 min at a time. Alive snails were brought to the laboratory, where their species were identified, according to their morphological features [27].

### Shedding and harvesting of cercariae

Snails were placed individually in 5 ml glass bottles filled to half their capacity with dechlorinated tap water and exposed to artificial light for about two hours to induce shedding of cercariae [28]. The water in each bottle was then examined under a dissecting microscope for the presence of cercariae. Snails that did not shed cercariae on the first exposure were kept in glass aquaria in the laboratory and rechecked for cercarial shedding, as described above, weekly for four weeks after collection. During this period, snails were fed lettuce and mortality among the snails was low.

### Identification of cercariae

Cercariae released by the snails were isolated, mounted on slides and stained with hematoxylin [28]. Morphologically cercariae types were identified based on gross characteristics, swimming behavior, resting position, and further cercarial development [28, 29]. Cercariae belonging to the genus *Schistosoma* (*S. haematobium* and avian schistosomes) were identified by their morphological features. *S. mansoni* cercariae were identified to species level based on morphology of adult worm and egg morphology recovered from mice infected experimentally with the cercariae [25].

### Environmental and physical characteristics

To explain variations in population densities of the freshwater snails, a number of environmental parameters were recorded during the study. Macrophyte cover and algal mass were scored visually and recorded as percentages of the area covered in the sampling sites. Temperature was measured using a mercury thermometer graduated from 0° to 50° centigrade graduation. Water depth was measured using a hard wooden ruler. Current velocity was measured by timing a floating object over a two - meter stretch. Water turbidity was visually graded into two categories, i.e. clear and turbid.

### Data analysis

Snail counts and prevalence of infections between seasons and years, based on simple descriptive statistics, were compared. To test associations between ecological factors (some of which were recoded; see Results) and densities of the most abundant snail species and their most prevalent infections, negative binomial regression [30] and logistic regression analysis [31] were conducted, respectively, adjusting for site. Using the same procedures, snail densities and prevalence rates of infections were compared between seasons and years, adjusting for site. *P*-values less than 0.05 were considered statistically significant.

## Results

**Fig. 2:**
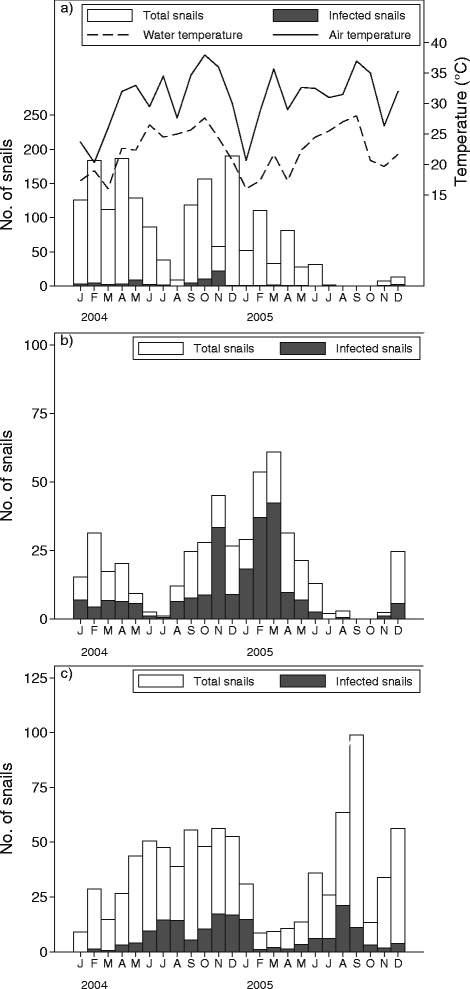
Density of snails (number of snails per standard search), infected snails, and mean water and air temperature, by month, from January 2004 to December 2005, in the East Nile locality, Khartoum, Sudan. **a** *Biomphalaria pfeifferi* infected with any trematodes, (**b**), *Bulinus truncatus* infected with xiphidiocercariae, (**c**) and *Melanoides tuberculata* infected with parapleurolophocercous cercariae

A total of 10,493 freshwater snails, belonging to seven species, were collected from the study area. The most abundant species was *B. pfeifferi*, constituting 48.6 % of the entire sample (see Table 1). Snail counts differed among these sites and also between years. For *B. pfeifferi* snails, the monthly arithmetic mean density in 2004 was 119.3 (see number of snails per standard search) and in 2005 it was 33.7; this difference was significant (see Table 2). The density of *B. truncatus* snails was comparable between the two years, i.e. 20.5 and 22.8 snails/standard search in 2004 and 2005, respectively. The density of *M. tuberculata* snails was 38.8 snails/standard search in 2004 and 28.4 in 2005. The density of *B. pfeifferi* snails tended to be lower during the summer months than in the winter months, i.e. 50.9 and 93.5 snails/standard search, respectively. The same was observed for *B. truncatus* snails (10.7 and 27.6, snails/standard search, respectively). For *M. tuberculata* snail, density was high during the summer, i.e. 43.6 and 28.5 during summer and winter, respectively. More detailed variations in the seasonal density of selected snail species are shown in Fig. 2.

**Table 1:** Total number of snails collected during monthly sampling in three irrigation canals etc

Species	Site 1		Site 2			Site 3				
	2004	2005	Total	2004	2005	Total	2004	2005	Total	All sites
*Biomphalaria pfeifferi*	401 (39.3)	2 (0.3)	403 (22.5)	2029 (62.5)	390 (44.5)	2419 (58.7)	1625 (57.9)	653 (36.9)	2278 (49.8)	5100 (48.6)
*Bulinus truncatus*	146 (14.3)	423 (54.9)	569 (31.8)	242 (7.5)	10 (1.1)	252 (6.1)	309 (11.0)	273 (15.4)	582 (12.7)	1403 (13.4)
*Bulinus forskalii*	4 (0.4)	14 (1.8)	18 (1.0)	165 (5.1)	333 (38.0)	498 (12.1)	6 (0.2)	2 (0.1)	8 (0.2)	524 (5.0)
*Lymnea natalensis*	161 (15.8)	93 (12.1)	254 (14.2)	67 (2.1)	2 (0.2)	69 (1.7)	0 (0.0)	6 (0.3)	6 (0.1)	329 (3.1)
*Physa acuta*	58 (5.7)	116 (15.0)	174 (9.7)	432 (13.3)	135 (15.4)	567 (13.8)	0 (0.0)	0 (0.0)	0 (0.0)	741 (7.1)
*Melanoides tuberculata*	146 (14.3)	39 (5.1)	185 (10.3)	306 (9.4)	6 (0.7)	312 (7.6)	867 (30.9)	836 (47.2)	1703 (37.2)	2200 (21.0)
*Cleopatra bulimoides*	105 (10.3)	84 (10.9)	189 (10.5)	5 (0.2)	1 (0.1)	6 (0.1)	1 (0.0)	0 (0.0)	1 (0.0)	196 (1.9)
Total	1021	771	1792	3246	877	4123	2808	1770	4578	10493

**Table 2:** Associations between snail counts for 3 snail species or infections in these and various factors tested one by one together with site so as to adjust for variation among sites

Factor (coding first category base)	Snail density (Count ratios)			Infections in snails (Odds ratios)			
	*Biomphalaria pfeifferi*	*Bulinus truncatus*	*Melanoides tuberculata*	*Schistosoma mansoni* (*B. pfeifferi)*	Xiphidio cercariae (*B. pfeifferi*)	Xiphidio cercariae (*B. truncatus*)	Parapleurolo-phocercous cercariae (*M. tuberculata*)
Year (2004 - 2005)	0.13***	0.85	0.30**	0.35	0.29*	1.43*	0.63***
Season (Summer - Winter)	1.74	2.61*	0.55	0.67	0.75	1.65**	0.95
Water temperature (°C)	0.92	0.97	1.09*	0.97	1.26***	0.98	0.98
Water temperature (previous month)	0.95	0.99	1.02	0.96	1.22***	1.16***	0.99
Depth (≤0.10 m - >0.10 m)	0.33*	0.81	0.32*	0.33	0.32***	1.28	0.63
Flow speed (≤0.40 m/s - >0.40 m/s)	2.24	1.02	3.05*	0.85	0.55***	0.61***	0.68
Turbidity (Clear-turbid)	1.09	0.49	1.84	1.52	0.61**	0.56**	1.13
Vegetation cover (≤40 % - >40 %)	2.26	1.39	0.89	3.12	3.98***	1.24*	1.30*

Numbers are exponentially transformed regression confidents from negative binomial regression on snail counts (count ratio) or from logistic regression on infection status of these snails (odds ratios)

*)*p* < 0.05; **)*p* < 0.01; ***)*p* < 0.001

**Fig. 3:**
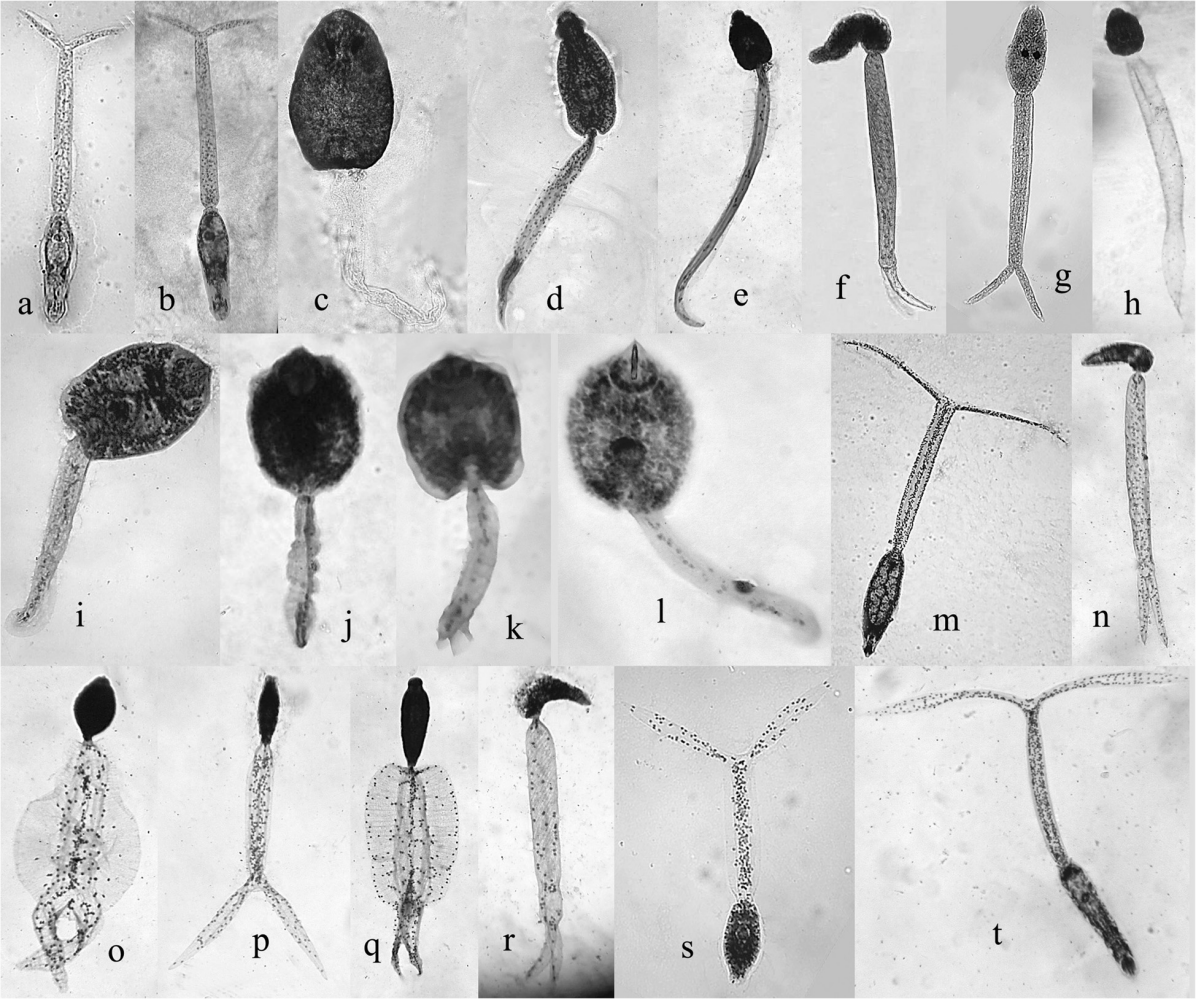
Morphotypes of cercariae recorded from five snail species in the East Nile locality, Khartoum, Sudan. The types are:- **a**) *Schistosoma haematobium* (or related species); **b**) *S. mansoni*; **c**) amphistome; **d**) echinostome; **e**) parapleurolophocercous; **f**) lophocercous-apharyngeate; **g**) avian schistosome, i.e. brevifurcate-apharyngeate distome cercariae; **h**) Type 1; **i** –**l**) four types of xiphidiocercariae: **i**) Type 1, **j**) Type 2, **k**) ornatae, **l**) armatae; **m**) LPM; **n**) Type 5; **o**) Type 2; **p**) Type 3; **q**) Type 4; **r**) Type 6; **s**) Type 7; **t**) longifurcate-pharyngeate distome cercaria (Strigea). The photos are not to the same scale

A total of 20 different types of cercariae were shed by five snail species (see Tables 3, 4, and Fig. 3); no infections were recorded in the *L. natalensis* and *P. acuta* snails. Xiphidiocercariae types 1 and 2 and another seven types (types 1, 2, 3, 4, 5, 6, and 7) of cercariae have not been previously described in Africa.

**Table 3:** Number of infections recorded in 5 different snail species collected from three irrigation canals

	Site			Snail species					
Cercariae type	Site 1	Site 2	Site 3	*Biomphalaria pfeifferi*	*Bulinus truncatus*	*Bulinus forskalii*	*Melanoides tuberculata*	*Cleopatra bulimoides*	Total
Xiphidiocercariae (4 types)	452 (82.8)	281 (90.6)	152 (24.2)	176 (80.7)	657 (95.9)	0	51 (9.9)	1 (1.6)	885 (59.6)
Parapleurolophocercous	24 (4.4)	2 (0.6)	410 (65.2)	0	0	0	436 (84.7)	0	436 (29.4)
Vivax (LPM)	60 (11.0)	2 (0.6)	1 (0.2)	0	0	0	0	63 (98.4)	63 (4.2)
Avian schistosome (BAD)	0	0	26 (4.1)	0	0	0	26 (5.0)	0	26 (1.8)
*S. mansoni*	1 (0.2)	14 (4.5)	9 (1.4)	24 (11.0)	0	0	0	0	24 (1.6)
Amphistome cercariae	6 (1.1)	7 (2.3)	4 (0.6)	0	17 (2.5)	0	0	0	17 (1.1)
Strigea (LPD)	0	0	8 (1.3)	7 (3.2)	1 (0.1)	0	0	0	8 (0.5)
Echinostome	0	1 (0.3)	5 (0.8)	4 (1.8)	2 (0.3)	0	0	0	6 (0.4)
Type 5	0	0	4 (0.6)	1 (0.5)	3 (0.4)	0	0	0	4 (0.3)
Type 7	0	0	4 (0.6)	1 (0.5)	2 (0.3)	1 (33.3)	0	0	4 (0.3)
*S. haematobium*	1 (0.2)	1 (0.3)	0	0	2 (0.3)	0	0	0	2 (0.1)
Type 1	0	2 (0.6)	0	0	0	2 (66.7)	0	0	2 (0.1)
Type 2	0	0	2 (0.3)	2 (0.9)	0	0	0	0	2 (0.1)
Type 3	0	0	2 (0.3)	2 (0.9)	0	0	0	0	2 (0.1)
Type 6	2 (0.4)	0	0	0	0	0	2 (0.4)	0	2 (0.1)
LA	0	0	1 (0.2)	0	1 (0.1)	0	0	0	1 (0.1)
Type 4	0	0	1 (0.2)	1 (0.5)	0	0	0	0	1 (0.1)
Total snails infected	546	310	629	218	685	3	515	64	885 (59.6)
Total types	7	8	14	9	8	2	4	2	17

**Table 4:** Measurements of the cercariae (μm)

Cercarial type	Body length	Tail stem	Tail furcae
*S. mansoni*	152	242	73
*S. haematobium*	140	227	73
Avian (BAD)	145	262	91
Amphistome	169	351	-
Echinostome	413	737	-
LA	78	293	56
Parapleurolophocercous	223	1048	-
Strigea (LPD)	157	212	201
Vivax (LPM)	140	200	150
Type 1	83	443	-
Type 2	80	194	161
Type 3	105	195	151
Type 4	159	248	131
Type 5	126	339	111
Type 6	68	194	57
Type 7	106	176	146
Armatae xiphidiocercariae	99	113	-
Ornatae xiphidiocercariae	96	106	-
Type 1 xiphidiocercariae	102	110	-
Type 2 xiphidiocercariae	67	92	-

Overall, regardless of species, 1484 snails were found to be infected (see Tables 3 and 4) with trematodes. The snail species with the highest prevalence of infection was *B. truncatus* (46.2 %). Double infections were recorded in only two *B. truncatus* snails and one *C. bulimoides* snail. The two *Bulinus* snails were found to be simultaneously shedding *S. haematobium* and xiphidiocercariae, whereas the *C. bulimoides* snail was found to be shedding xiphidiocercariae and longifurcate-pharyngeate monostome cercariae Vivax (LPM) cercariae at the same time.

The most common type of cercariae recovered from the study area was xiphidiocercariae type 1 from *B. truncatus* snails, which accounted for 44.3 % of all infections (see Tables 3 and 4 and Fig. 2). This was followed by parapleurolophocercous (29.4 % of all infections) and LPM (4.2 % of all infections). *Schistosoma mansoni* cercariae represented 1.6 % of all infections recorded, while *S. haematobium* (or related species) was recovered from only two *B. truncatus* snails (0.1 %). The avian schistosome cercariae revealed a prevalence of 1.8 %. Ornatae and armatae xiphidiocercariae contributed to 11.9 % and 3.4 % of all infections, respectively.

It was observed that *B. pfeifferi* snails were hosts for diverse cercarial fauna (nine types), with an overall prevalence of infection of 14.7 % (see Tables 3 and 4 and Fig. 2). Meanwhile *B. truncatus* snails served as hosts for eight types of cercariae (see Tables 3 and 4). *M. tuberculata* snails shed four types of cercariae and contributed to 34.7 % of all infections. A total of 24 specimens of *B. pfeifferi* snails were found to be infected with *S. mansoni*, with the prevalence of infection not differing significantly between years and seasons (see Table 2). The odds of finding xiphidiocercariae in *B. pfeifferi* snails in 2005 was 0.25 (*p* < 0.001) of that in 2004, while the prevalence of infection did not differ between seasons (see Table 2). The maximum monthly overall prevalence (three sites combined) of xiphidiocercariae infection in *B. pfeifferi* snails was 37.4 (number of snails collected was 174). Only two specimens of *B. truncatus* snails were found to be infected with schistosomes. The odds of finding xiphidiocercariae in *B. truncatus* snails in 2005 was 1.51 (*p* < 0.001) of that in 2004, and the odds of infection during winter was 1.62 (*p* < 0.001) of that during summer months (see Table 2). The maximum monthly overall prevalence of xiphidiocercariae infection in *B. truncatus* snails was 74.1 % (number of snails collected was 135). The odds of finding parapleurolophocercous cercariae in *M. tuberculata* snails did not differ significantly between years and seasons. The maximum monthly overall prevalence of parapleurolophocercous cercariae (see Fig. 2) in *M. tuberculata* snails was 47.3 % (number of snails collected was 93).

The mean monthly water temperature (mean of three sites) ranged from 22.3 °C to 28.0 °C in the summer, and from 16.0 °C to 27.7 °C in the winter. The air temperature ranged from 27.7 °C to 37.0 °C in the summer, and from 20.3 °C to 38.0 °C in the winter. Only the density of *M. tuberculata* snails was associated with water temperature (see Table 2): the odds of finding xiphidiocercariae in snails increased with higher water temperature, as well as with water temperature from the previous month. Monthly mean water depth varied significantly in both seasons, i.e. from 0.3 to 43 cm during the entire period. Monthly mean water velocity ranged from 0.2 to 0.9 m/s in the summer, and from 0.6 to 1.2 m/s in the winter. Water turbidity records varied; water was clear in April and generally turbid in October. Water depth, current speed, and turbidity are linked; generally, coverage by aquatic macrophytes ranged from 10– 75 % in both seasons. High coverage by aquatic vegetation was associated with higher odds of finding infected snails (see Table 2).

## Discussion

This study found a generally high density of snails in the irrigation canals studied, as well as a diverse trematode fauna, with 20 morphotypes of cercariae identified, some of which might represent more than one species. The prevalence of infection in some snail species is high. The most common snail species found was *B. pfeifferi* and although the numbers of snails infected with *S. mansoni* are relatively few, this level of infection seems to be sufficient to maintain intense transmission to humans, as schistosomiasis is perceived as a major problem in the human host. This is in line with earlier findings [32]. In a previous study undertaken in this area (from 2003 to 2004), it was noted that although a high percentage of school children were infected with *S. haematobium* and *S. mansoni*, there were very few snails infected with these trematodes in the adjacent irrigation canals [26].

Although the design of these irrigation systems is similar to that of the Gezira-Managil scheme, it is noteworthy that minor canals in our study area do not support schistosome intermediate hosts, which is in contrast with the Gezira irrigated area, where minor canals constitute the most important transmission sites [13, 20].

It was observed in this study that the density of snails varied over the years. Usually in intermittent irrigation systems, snail density is greatly affected by irrigation practices and canal maintenance. Thus, the speed of the water in the *abueshreens* may be high during the initial period after an irrigation cycle has begun and this may dislodge snails that are then carried downstream. Canal maintenance such as vegetation or mud removal contributes to the removal of the snails and thus greatly influences the size of snail populations. Therefore, the effect of other potential factors, such as seasonal temperature fluctuations and growth of aquatic macro- and -micro-flora, may be difficult to assess.

Prevalence of trematode infections in snails is high in this area, and this is clearly an important factor for snail population density fluctuations and transmission of schistosomes through antagonism between trematode species or direct pathological effects of trematodes on snails. In the Gezira irrigation system, it was noted that larval echinostomes are abundant in local populations of *B. truncatus* snails, a factor that may to some extent be responsible for the uneven distribution and low prevalence of *S. haematobium* in the region [33].

Larval trematodes may act as regulators of snail populations if prevalence of infection in natural snail populations is high [34, 35]. It is known that certain trematodes may, in some cases, be responsible for the elimination of snail populations [1]. Some of these species might be manipulated to achieve biological control of snail-transmitted diseases [36, 37]. Larval trematode infections can also be used as bio indicators of environmental quality [38, 39], in that a change in species richness and prevalence of infection over time may reflect environmental change.

Double infections in snails are rare in this area, and this has been attributed to inter-species antagonism [40, 41]. Possibly, the low prevalence of *S. haematobium* infections in *B. truncatus* snails in the present study could be due to antagonism by xiphidiocercariae, which were found with high rates. Sousa [42] also speculated that double infections could be more pathogenic when compared to single species infections. As a result, snails with multiple infections face higher chances of mortality compared to those with single infections, and may therefore be underrepresented in snail collections. However, temporal and spatial variations in the abundance of eggs and miracidia of different trematode species have been considered to be more important in determining how often a snail is simultaneously infected by two or more species or how often established infections are challenged [42–46].

Whether the diverse trematode fauna also reflect high diversity of potential final hosts, both aquatic and terrestrial species, remains to be studied. Loss of biodiversity could adversely affect human health, with multiple potential reasons for this [47–50]

No infections were found in *L. natalensis* and *P. acuta* snails in this study, however this is in contrast to other studies. For example, it was found that *L. natalensis* was the most important intermediate snail host for transmitting a wide variety of trematodes in Tanzania [1]. Many records in Egypt pointed to *P. acuta* being the most infected snail in the freshwater snail community [51, 52]. Resistance of snails to trematode infections has been reported to play a role in determining prevalences rates of infection [53]. However, low numbers of snails with larval trematodes were recorded in the present study, and it has been reported that prevalence of larval trematode infections are dependent on snail numbers [54–56].

With the exception of brevifurcate apharyngeate monostome cercaria, the cercarial types reported by Chingwena et al. [57] in Zimbabwe were all recorded in the present study, in addition to new ones. Amphistome cercariae were recorded in *B. tropicus*, *B. globosus*, *B. forskalii*, and *B. pfeifferi* snails in Zimbabwe [57]. In the present study, these cercariae were recovered only from *B. truncatus* snails. Results from the present study suggest that *B. pfeifferi* and *B. truncatus* snails may be major hosts for a variety of larval trematodes, as they together harbor 15 types of trematode cercariae. However *B. tropicus* was the most important intermediate snail host for transmitting a wide variety of trematode species in Zimbabwe [57].

This study demonstrated that transmission of trematodes in this area and the prevalence of infection in the snail hosts are very intense. In order to explain the observed distribution patterns further studies are required to identify final hosts for most of these species and to further explore the possibility that these trematodes can be augmented so as to either affect snail populations directly or prevent transmission of trematodes of medical and/or veterinary importance.

## Conclusion

Besides schistosome species, several other larval trematodes are prevalent. Especially xiphidiocercariae, were very prevalent in *B. truncatus* snails. Since these trematodes cause mortality among snails and possibly compete with schistosomes within intermediate hosts (antagonism), we suggest that further studies be undertaken to test whether prevalence of some of these other trematode could be augmented to an extent where they could be used in the biological control of schistosomiasis

## Additional file

Additional file 1:
**Multilingual abstracts in the six official working languages of the United Nations.** (PDF 383 kb)

## Abbreviation

LPM: longifurcate-pharyngeate monostome cercariae Vivax
