# Supplementary material for: Types of trematodes infecting freshwater snails found in irrigation canals in the East Nile locality, Khartoum, Sudan
Source: Infect Dis Poverty. 2016 Feb 25;5:16. doi: 10.1186/s40249-016-0108-y (PMC4766606; doi:10.1186/s40249-016-0108-y)
Supplement: Additional file 1: — Multilingual abstracts in the six official working languages of the United Nations. (PDF 383 kb) [file 40249_2016_108_MOESM1_ESM.pdf]

Translation of the abstract into the six official working languages of the United Nation

أنواع الديدان المثقوبة التي تصيب القواقع في المياه العذبة المتواجدة في قنوات الري في محلية شرق النيل، الخرطوم، السودان

نضال أ. أ. محمد، هنري مادسن، وعبد العزيز أ. ر. م. أحمد

ملخص

خلفية: تم دراسة قواقع في المياه العذبة من جنسي *Biomphalaria* و *Bulinus* باستفاضة نظرا للدور الذي تقوم به كعائل وسيط للبلهارسيا. وفي السودان على وجه التحديد، ركزت معظم الدراسات على المكافحة الكيميائية والبيئية للجنسين، ولكن القليل من الدراسات تناولت المكافحة البيولوجية لهما. بحثت هذه الدراسة التعايش بين الأنواع الأخرى من القواقع في المياه العذبة وهذين الجنسين جنبا إلى جنب مع داء المثقوبات فيما يتعلق بعدد من العوامل البيئية في محلية شرق النيل بولاية الخرطوم، السودان.

الأساليب: أخذت عينات من القواقع في المياه العذبة من قنوات الري (abueshreens) شهريا خلال الفترة من يناير 2004 إلى ديسمبر 2005. تم فحص القواقع لداء المثقوبات من قبل ظهور المذنبات مباشرة بعد الجمع ثم أسبوعيا لمدة أربعة أسابيع إضافية للسماح بنضوج العدوى سابقة للظهور. وسجل أيضا الغطاء النباتي في مواقع الدراسة فضلا عن الخصائص الفيزيائية والكيميائية للمياه، بما في ذلك درجة الحرارة.

النتائج: تم جمع ما مجموعه 10493 قوقعة تمثل سبعة أنواع. وكانت الأنواع الأكثر وفرة هي *Biomphalaria pfeifferi*، وهو ما يمثل 48.6٪ من العينة. وعموما، وجد أن 14.1٪ من القواقع تنتج نوعا من السركاريا. تم العثور على خمسة أنواع من داء المثقوبات. من بين هؤلاء وجد أن نوع *Bulinus truncatus* هو الأكثر إصابة، بمعدل انتشار 46.2٪. وسجلت إصابات مزدوجة في اثنين فقط من القواقع *B. truncatus* وواحدة من قواقع *Cleopatra bulimoides*. وسجل عشرين نمط شكلي مختلف من السركاريا، سبعة منها لا يبدو متواقا مع السركاريا الموصوفة سابقا من أفريقيا. كان النوع 1 من Xiphidiocercariae هو الأكثر شيوعا من السركاريا المعثور عليها، وهو ما يمثل 44.3٪ من مجموع الإصابات. تميل كثافة القواقع إلى أن تكون أقل خلال أشهر الصيف عن أشهر الشتاء، ما عدا قواقع *M. tuberculata*، التي لم تتأثر بالتغيرات الموسمية.

الخلاصة: تشير نتائج هذه الدراسة إلى أنه إلى جانب البلهارسيا، تم العثور على يرقات الديدان المثقوبة الأخرى، وبعضها يستخدم نفس العائل الوسيط مثل البلهارسيا. وينبغي إجراء مزيد من الدراسات لتحديد ما إذا كان بعض هذه الديدان المثقوبة يمكن إحداث تعديلات عليها للمكافحة البيولوجية لمرض البلهارسيا.

Translated from English version into Arabic by Mahmoud Sami, through

TRANSLATORS
WITHOUT BORDERS

苏丹喀土穆东尼罗河地区灌溉水渠感染淡水螺的吸虫种类

Nidal A.I. Mohammed, Henry Madsen, and Abdel Aziz A.R.M. Ahmed

摘要

引言：隶属于扁卷螺科（Planorbidae）的双脐螺属（*Biomphalaria*）和水泡螺属（*Bulinus*）两个属的淡水螺作为血吸虫病的中间宿主，得到了广泛研究。在苏丹，大多数研究尤其关注这两个属螺种的化学和生态学控制，但少有从生物学角度来研究的。本文研究了苏丹尼罗河东岸流域喀土穆州上述两属螺类与其他淡水螺的共生情况，以及环境因素的影响。

方法：2004 年 1 月至 2005 年 12 月，每月在灌溉渠（农渠）中采集淡水螺。收集后立即采用逸蚴法检查淡水螺感染吸虫情况。对于首次逸蚴试验未发现尾蚴的淡水螺继续培养四周，每周检查淡水螺感染情况一次。同时记录研究区域的植被覆盖、水流的物理化学特性（包括温度）等。

结果：共采集到 10,493 个淡水螺，分属七个种。其中数量最多的是菲氏双脐螺（*Biomphalaria pfeifferi*），

占 48.6%。14.1%的淡水螺有尾蚴逸出。五种淡水螺感染尾蚴。其中，截形水泡螺（*Bulinus truncatus*）感染最为严重，感染率达 46.2%。两个截形水泡螺和一个 *Cleopatra bulimoides* 出现双重感染。共记录了 20 种不同形态的尾蚴，其中七种与先前非洲的记录不符。1 型剑口尾蚴是所见尾蚴中最常见的类型，占总数的 44.3%。除了不受季节变换影响的瘤拟黑螺（*Melanoides tuberculata*），夏季螺类密度比冬季低，。
结论：本研究结果表明，除了血吸虫，还发现其他吸虫幼虫，有些吸虫与血吸虫共用中间宿主（螺类）。这些吸虫是否可用于血吸虫病的生物控制还有待于进一步研究。

Translated from English version into Chinese by Miao Junling, edited by Yang Pin, through

Types de trématodes infestant des escargots d'eau douce identifiés dans des canaux d'irrigation dans la localité du Nil oriental, Khartoum, Soudan

Nidal A.I. Mohammed, Henry Madsen et Abdel Aziz A.R.M. Ahmed

Résumé
Contexte: Les planorbes, des escargots d'eau douce, de deux genres différents *Biomphalaria* et *Bulinus* ont fait l'objet d'études approfondies en raison de leur rôle en tant qu'hôte intermédiaire de la schistosomiase. La plupart des études, notamment au Soudan, se sont concentrées sur le contrôle chimique et écologique de ces deux genres, mais peu d'études ont été menées pour analyser leur contrôle biologique. La présente étude a exploré la coexistence d'autres espèces d'escargots d'eau douce et des deux genres porteurs d'une infection à trématodes en rapport avec un certain nombre de facteurs environnementaux dans la localité du Nil oriental, État de Khartoum, Soudan.
Méthodes: Des escargots d'eau douce ont été collectés chaque mois dans des canaux d'irrigation (*abueshreens*) de janvier 2004 à décembre 2005. Un examen a été réalisé sur les escargots afin d'y déterminer la présence d'une infection à trématodes par émergence de cercaires immédiatement après la collecte, puis à un rythme hebdomadaire pendant quatre semaines supplémentaires afin de permettre la maturation des infections prépatentes. La couverture végétale sur les sites d'étude ainsi que les caractéristiques physicochimiques de l'eau, y compris sa température, ont aussi fait l'objet d'observations.
Résultats: Au total, 10 493 escargots représentant sept espèces différentes ont été collectés. L'espèce *Biomphalaria pfeifferi* était la plus abondante et représentait 48,6 % de l'échantillon. Au total, 14,1 % des escargots étaient infestés par certains types de cercaires. Nous avons pu constater que cinq espèces étaient atteintes d'infections et parmi ces dernières, l'espèce *Bulinus truncatus* était la plus fortement infectée avec une prévalence globale de 46,2 %. Des infections doubles ont été observées chez uniquement deux escargots *B. truncatus* et un escargot *Cleopatra bulimoides*. Vingt types morphologiques différents de cercaires ont été déterminés, sept d'entre eux ne semblaient pas correspondre aux cercaires d'Afrique déjà décrits. La xiphidiocercaire de type 1 constituait le type le plus courant de cercaire identifié et était à l'origine de 44,3 % de l'ensemble des infections. La densité des escargots avait tendance à être plus faible au cours des mois d'été que pendant les mois d'hiver, à l'exception des escargots *M. tuberculata* qui n'étaient pas affectés par les changements de saison.
Conclusion: Les résultats de la présente étude indiquent la présence d'autres trématodes larvaires en plus des schistosomes et démontrent que certains trématodes utilisent les mêmes hôtes intermédiaires que les schistosomes. D'autres études doivent être réalisées afin de déterminer si certains de ces trématodes pourraient être manipulés à des fins de contrôle biologique de la schistosomiase.

Translated from English version into French by eric ragu, through

Типы трематодов, заражающих пресноводных улиток, найденных в ирригационных каналах в районе Восточного Нила, Хартум, Судан

Nidal A.I. Mohammed, Henry Madsen и Abdel Aziz A.R.M. Ahmed

Резюме

Основание: Пресноводные улитки “катушки”двух видов, *Biomphalaria* и *Bulinus* были подвергнуты активному изучению, так как они являются промежуточными носителями шистосомиаза. В Судане, в частности, большинство исследований сфокусировано на химическом и экологическом контроле этих двух видов, и лишь небольшое количество исследований посвящено биологическому контролю. Это исследование освещает сосуществование других видов пресноводных улиток и и этих двух видов, переносящих трематодов, и связи с несколькими факторами окружающей среды в районе Восточного Нила, Хартум, Судан.

Методы: Пресноводные улитки из ирригационных каналов (*abueshreens*) выбирались ежемесячно с января 2004 по декабрь 2005. Анализ улиток на наличие инфекции трематодов методом выделения церкарий производился сразу же после забора, а затем еженедельно в течение дополнительных четырех недель, для вызревания препатентной инфекции. Также регистрировались: растительноый покров местности, физикохимические характеристики воды, включая температуру.

Результаты: Всего было собрано 10 493 улитки, представляющие 7 видов. Самый многочисленный вид - *Biomphalaria pfeifferi*, представляющий 48,6% выборки. В целом, на 14,1% улиток был обнаружен какой-либо тип церкарий. Пять видов улиток оказались носителями инфекций; среди них наиболее зараженными оказались представители вида *Bulinus truncatus*, общее распространение составило 46,2%. Два вида инфекции одновременно было обнаружено только у двух улиток *B. truncatus* и одной улитки *Cleopatra bulimoides*. Было зарегистрировано двадцать разных видов морфотипов, семь из которых не совпадают с ранее описанными церкариями в Африке. Самым распространенным видом церкариев оказался Xiphidiocercariae тип 1 - 44,3% всех инфекций. Плотность улиток имеет тендецнию уменьшаться в летние месяцы по сравнению с зимними месяцами, кроме вида *M. tuberculata*, эти улитки не подвержены смене сезонов.

Вывод: Это исследование показало, что помимо шистосомиаза были обнаружены и другие виды личиночных трематодов, некоторые из них пользуются теми же промежуточными носителями, что и шистосомиаз. Должны быть произведены дальнейшие исследования, с целью показать, можно ли манипулировать этими трематодами для контроля шистосомиаза биологическим путем.

Translated from English version into Russian by Anna Philippova, through

Tipos de tremátodos que infectan a los caracoles de agua dulce hallados en canales de regadío en la localidad de Nilo Este (Jartum, Sudán)

Nidal A.I. Mohammed, Henry Madsen y Abdel Aziz A.R.M. Ahmed

Resumen

Información de referencia: Los caracoles planórbidos de agua dulce de los dos géneros, *Biomphalaria* y *Bulinus* se han estudiado a fondo debido al importante papel que desempeñan como huéspedes intermedios de la esquistosomiasis. En Sudán concretamente, la mayoría de estudios se han centrado en el control químico y ecológico de los dos géneros, pero se han dedicado pocos estudios a su control biológico. Este estudio exploró la coexistencia de otras especies de caracoles de agua dulce y los dos géneros, junto con sus infecciones de tremátodos, y su relación con diversos factores medioambientales en la localidad de Nilo Este (Jartum, Sudán).

Métodos: Se realizaron muestreos mensualmente de los caracoles de agua dulce de canales de regadío (*abueshreens*) entre enero de 2004 y diciembre de 2005. Los caracoles se examinaron en busca de infecciones de tremátodos por aparición de cercarias inmediatamente después de su recogida y luego semanalmente durante cuatro semanas más para permitir la madurez de las infecciones prevalentes. También se registraron datos sobre la cobertura vegetal en los lugares estudiados, así como sobre las características fisicoquímicas del agua, como la temperatura.

Resultados: Se recogieron en total 10.493 caracoles de siete especies. La especie más abundante fue *Biomphalaria pfeifferi*, que representó un 48,6% de las muestras. En total, se averiguó que un 14,1% de los caracoles arrojaban algún tipo de cercaria. Otras cinco especies tenían infecciones, y entre ellas se observó que la especie *Bulinus truncatus* era la que estaba más infectada, con una presencia total del 46,2%. Se registraron infecciones dobles en tan solo dos caracoles *B. truncatus* y en un caracol *Cleopatra bulimoides*. Se registraron veinte morfotipos diferentes de cercaria, siete de los cuales no parecían coincidir con las cercarias descritas anteriormente en África. La Xiphidiocercariae de tipo 1 fue el tipo de cercaria más común que se ha recuperado al representar el 44,3% de todas las infecciones. La densidad de caracoles tendía a ser más baja en los meses de verano que en los meses de invierno excepto para los caracoles *M. tuberculata*, que no se vieron afectados por cambios estacionales.

Conclusión: Los hallazgos de este estudio indican que además de la esquistosomiasis se encontraron otras larvas de trematodo y algunos utilizan el mismo huésped intermedio que los esquistosomas. Se deberían llevar a cabo otros estudios para determinar si algunos de estos tremátodos se pueden manipular para el control biológico de la esquistosomiasis.

Translated from English version into Spanish by SergioLorenzi, through

TRANSLATORS
WITHOUT BORDERS
